# Generative adversarial networks for anonymous acneic face dataset generation

**DOI:** 10.1371/journal.pone.0297958

**Published:** 2024-04-16

**Authors:** Hazem Zein, Samer Chantaf, Régis Fournier, Amine Nait-Ali

**Affiliations:** 1 LISSI Laboratory, Université Paris-Est Créteil, Vitry-sur-Seine, France; 2 Faculty of Technology, Lebanese University, Saida, Lebanon; Karunya Institute of Technology and Sciences, INDIA

## Abstract

It is well known that the performance of any classification model is effective if the dataset used for the training process and the test process satisfy some specific requirements. In other words, the more the dataset size is large, balanced, and representative, the more one can trust the proposed model’s effectiveness and, consequently, the obtained results. Unfortunately, large-size anonymous datasets are generally not publicly available in biomedical applications, especially those dealing with pathological human face images. This concern makes using deep-learning-based approaches challenging to deploy and difficult to reproduce or verify some published results. In this paper, we propose an efficient method to generate a realistic anonymous synthetic dataset of human faces, focusing on attributes related to acne disorders at three distinct levels of severity (Mild, Moderate, and Severe). Notably, our approach initiates from a small dataset of facial acne images, leveraging generative techniques to augment and diversify the dataset, ensuring comprehensive coverage of acne severity levels while maintaining anonymity and realism in the synthetic data. Therefore, a specific hierarchy StyleGAN-based algorithm trained at distinct levels is considered. Moreover, the utilization of generative adversarial networks for augmentation offers a means to circumvent potential privacy or legal concerns associated with acquiring medical datasets. This is attributed to the synthetic nature of the generated data, where no actual subjects are present, thereby ensuring compliance with privacy regulations and legal considerations. To evaluate the performance of the proposed scheme, we consider a CNN-based classification system, trained using the generated synthetic acneic face images and tested using authentic face images. Consequently, we show that an accuracy of 97.6% is achieved using InceptionResNetv2. As a result, this work allows the scientific community to employ the generated synthetic dataset for any data processing application without restrictions on legal or ethical concerns. Moreover, this approach can also be extended to other applications requiring the generation of synthetic medical images.

## 1 Introduction

Skin diseases occur in people of different ages and cultures and they are more common than other diseases worldwide [1] and can be caused by many factors such as diet, hormones, virus, and bacteria. There are various types of skin diseases like acne, eczema, rosacea, psoriasis, vitiligo. A study conducted in 2017 showed that dermatologists density in the United States is 3.4 per 100000 persons which is still lower than the needed density [2]. Deep Learning provides the ability to improve the work of dermatologist with an AI assisted diagnosis system which lowers the burden on dermatologists. Deep Learning has been proven to yield great results on many image classification tasks including dermatology applications, and it can help classify skin diseases that are highly similar to each other, but in the dermatology field we face multiple obstacles before collecting data needed to feed deep learning models such as unavailability of public datasets, privacy and legal issues, low quality datasets: datasets focused only on some types of skin diseases. Generative adversarial networks are the solution to skip all these obstacles and present a way to augment our data with artificial images. GANs belong to the generative modeling type. GANs learn to generate synthetic samples indistinguishable from real ones after being trained on real samples. Many published research papers have used GANs in the generation of skin disease images and CNNs to classify skin disease types. In 2018 Baur et. al. used DCGAN and LAPGAN on ISIC2018 dataset to generate synthetic images of skin lesions [3]. In 2020 Ghorbani et al. proposed a model based on Pix2Pix architecture to generate images of skin lesions with a resolution of 256x256. Their dataset consisted of 49920 high resolution images [4]. Xiang et. al. in 2020 trained AC-GAN using HAM10000 containing skin lesions images with a resolution of 64x64 upscaled to 256x256 to train multiple classification models for result comparison [5]. Junayed et. al. proposed a CNN architecture “AcneNet” which was trained on a dataset of 5 classes of acne containing 360 images per class with a resolution of 224x224. AcneNet reached an accuracy of 99.44% in one class and a minimum of 94% for the rest of the classes [6]. Wu et. al. (2019) trained different CNN models on a dataset of 6 classes of skin diseases and a total of 2656 images. The best model achieved 77% average precision [7]. In 2021, Srinivasu et. al. used MobileNetV2 and LSTM that were trained on HAM10000 skin lesions dataset and they achieved an accuracy of 85.34% [8]. Zhao et. al. (2021) used ISIC2019 skin lesions dataset augmented with a modified architecture of StyleGAN that outputs images with a resolution of 224x224 to train DenseNet201 for skin lesions classification [9]. La Salvia et. al. (2022) used DCGAN to generate hyperspectral skin lesion images. DCGAN was trained with 5000 RGB images (50x50x3 resolution) from HAM10000 then hyperspectral images (acquired from two hospitals) were used to apply transfer learning on the GAN model (50x50x116 resolution). Resnet18 was trained on synthetic data only and scored an accuracy above 80% [10]. In this paper we propose using generative adversarial networks to give the ability to the medical community to generate datasets of realistic artificial faces with acne disease and to remove the barriers on access to data by generating highly realistic synthetic images that can allow us to reach same results as when using real images. To prove that the generated images can replace real images in deep learning applications, we trained three convolutional neural networks: InceptionResNetV2, ResNet50V2 and ResNet152V2 only on synthetic images and testing was done on real facial acne images not included in the training process. This paper is structured as follows: In section 2 we present the methodology including dataset gathering, algorithms and preprocessing steps to reach our objectives. In section 3 we discuss all the results obtained and finally we conclude this paper in Section 4.

## 2 Materials and methods

In recent years, deep learning is being implemented more in skin disease applications due to its high accuracy in classifying diseases compared to traditional methods. Subsection 2.1 and 2.2 give a brief overview on convolutional neural networks and generative adversarial networks and present the different deep learning models used in this paper. In subsection 2.3 we discuss the methodology used in this paper.

### 2.1 Convolutional Neural Networks

Convolutional Neural Networks belong to the class of artificial neural networks and their accuracy has surpassed traditional methods and specifically when the data is images. It is the most popular and mostly used among deep learning algorithms. CNNs are composed of an input layer multiple hidden layers and an output layer and have multiple types of layers including convolutional layer, pooling layer and fully-connected layer. CNNs are being used in many applications like object recognition, image classification, natural language processing [11]. In this Paper we used 3 pre-trained CNN Models:

- InceptionResNetV2 is a hybrid of Inception networks and Residual Connections without filter concatenation but it comes with a heavier computational cost [12],
- ResNet152V2 and ResNet50V2 are two models with high number of layers as ResNet152V2 has 152 layers and ResNet50V2 has 50 layers. The main difference between ResNet V1 and V2 is that in V2 batch normalization and relu activation are used before each weight layer [13].

### 2.2 Generative adversarial networks

**Fig 1:**
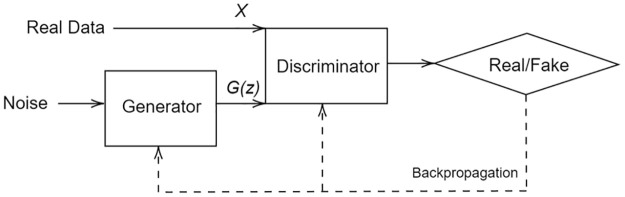
GAN diagram composed of two models: Generator and discriminator. Backpropagation is used to minimize the loss of the two models.

GANs are composed of two models as shown in Fig 1: generator and discriminator. The generator’s role is to generate synthetic samples to fool the discriminator into classifying these samples as real. The discriminator classifies data as either coming from original dataset or from the generator’s output. Both models are competing to improve each other and using backpropagation to reduce each model’s loss by adjusting the weights of each model depending on the impact on the output [14].

#### 2.2.1 StyleGAN2

StyleGAN developed by NVIDIA in 2018, brings the following improvements to the generator model architecture:

- Baseline Progressive GAN.
- Tuning and bilinear up/down sampling
- Addition of mapping, styles and adaptive instance normalization.
- Removal of latent vector input to generator.
- Noise addition at each block of the generator.
- Addition Mixing regularization [15].

**Fig 2:**
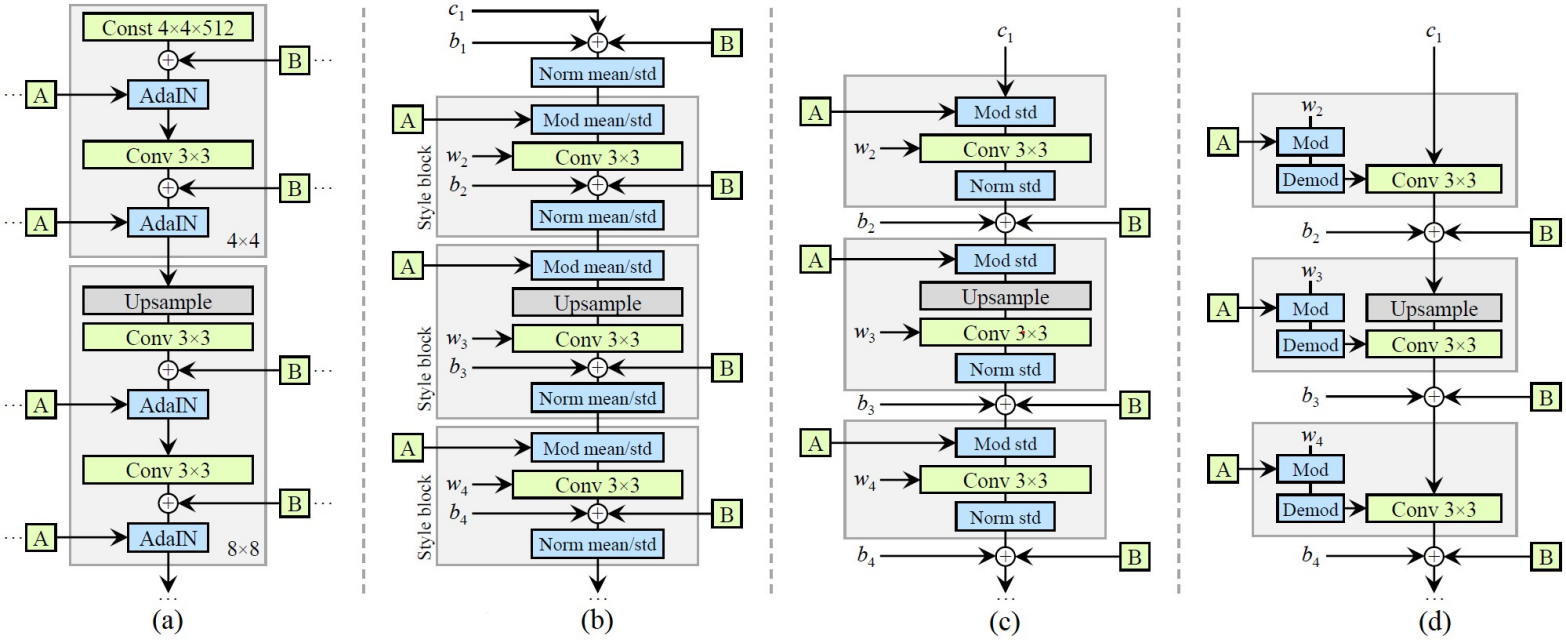
Comparison of StyleGAN and StyleGAN2 architecture. Moving from the use of adaptive instance normalization in StyleGAN to using weight demodulation in StyleGAN2 and shifting the addition of noise to the outside of generator blocks: (a) StyleGAN Architecture. (b) StyleGAN detailed. (c) StyleGAN Revised Architecture. (d) Weight demodulation [15, 16].

Fig 2(a) and 2(b) show the architecture of the first StyleGAN and part (c) is the result of switching to a form that does not use AdaIN (Adaptive Instance Normalization). The same operation as normalization by AdaIN is performed by an operation called Weight demodulation (dividing the weight of the conv layer by the standard deviation) [16].

#### 2.2.2 StyleGAN2 advantages compared to other GANs

- High quality image synthesis: StyleGAN2 achieves superior image synthesis quality compared to its predecessor and other generative models by integrating advancements in architecture and training strategies. Key features include a mapping network for enhanced style control, adaptive instance normalization (AdaIN) facilitating nuanced appearance adjustments, and the two-timescale update rule (TTUR) for maintaining balanced training dynamics. Path length regularization promotes smooth variations in the latent space, while data augmentation improves model generalization. The model’s refined noise injection mechanism and emphasis on diverse transformations contribute to the generation of high-quality and realistic images, establishing StyleGAN2 as a prominent approach in generative image synthesis [15, 16].
- Image style control: StyleGAN2 employs a network for mapping styles that transforms a latent code into a style vector. The idea behind interpretable directions within the latent space suggests that specific orientations are associated with meaningful semantic changes in the resulting images. this vector can be used to modify aspects of the images (Example: hair color, adding glasses, head position) [17, 18]
- Higher output resolution (1024x1024) [16] compared to other GANs. Example: BigGAN (512x512) [19], DCGAN (64x64, 256x256 and 512x512) [20, 21], LAPGAN (64x64) [22].

### 2.3 Methodology

**Fig 3:**
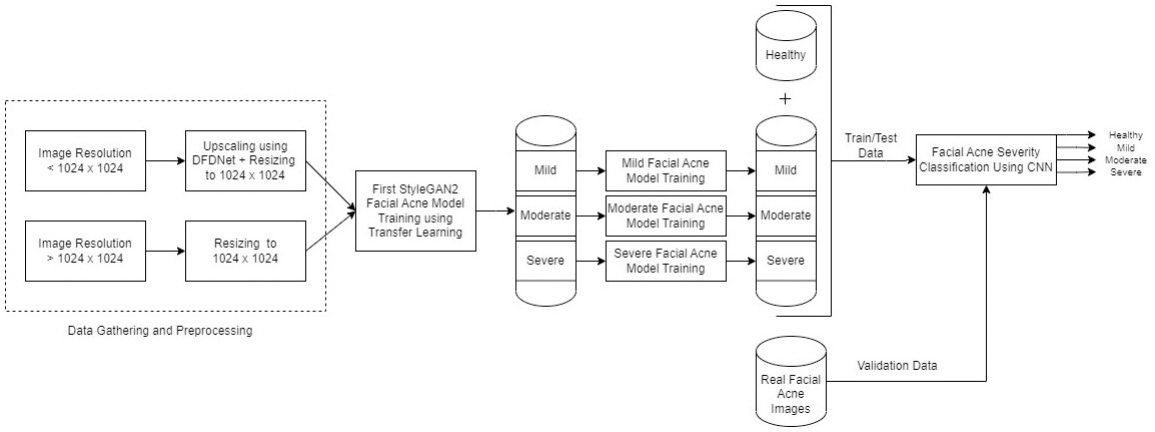
Block diagram representing the methodology. First step is gathering facial acne images from multiple sources, Second data processing to be compatible with the GAN input layer, then training StyleGAN2 models with different levels of facial acne severity using transfer learning and sorting the output images into 3 classes: mild, moderate and severe and adding a synthetic healthy class. Finally training a CNN model on the previous synthetic dataset to evaluate its performance on real images.

**Fig 4:**
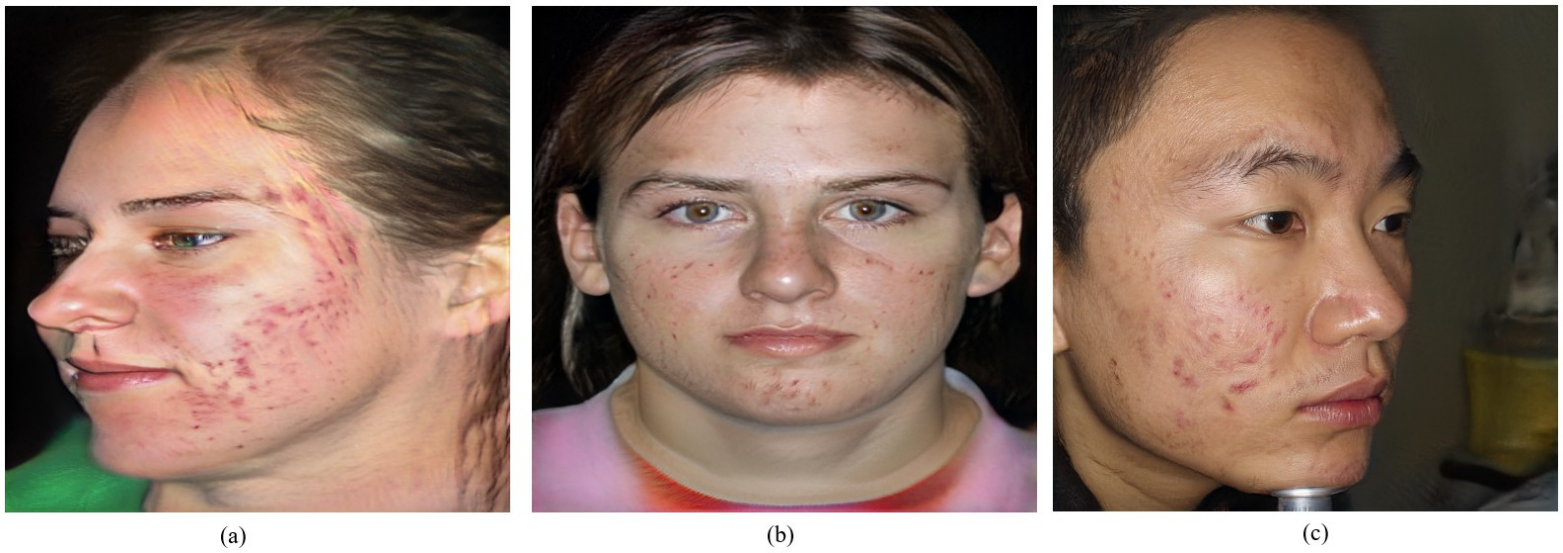
Synthetic dataset cleaning. (a) Deformed image (b) facial acne images with visible artifacts (c) Correct facial acne image. Images same as (a) and (b) are removed from the dataset.

In this paper, we followed the methodology shown in Fig 3 to reach our objective. We used style based generative adversarial network 2 (StyleGAN2) to generate our datasets of facial acne disease due to the high resolutions it can reach (1024x1024) and highly realistic results. Although we collected 1473 images of different facial acne severities, this dataset was not enough for training 3 GANs to generate each level of severity (Mild, Moderate, Severe). To overcome this problem, we proceeded with the following steps:

1. **Data Gathering**: First source of images is ACNE04 [23] dataset. This dataset consists of 4 levels of facial acne severity labeled by dermatologists reaching a total of 1073 images: Low severity(Level 0) with 418 images, Medium severity (level 1) contains 526 images and high severity (Level 2) has 129 images. The resolution of images in the Acne04 is 3112x3456. 400 facial acne images with different levels of severity were gathered from google images with low resolutions.
2. **Data pre-processing**: In this work, we used two data preprocessing methods, images in the orignal dataset were either resized or upscaled to finally build a dataset compatible with StyleGAN2 input size.
  - **Resizing:** StyleGAN2 requires that the images resolution on the input to be equal to 1024x1024, resizing each input is required. Specifically, if the input is oversized, simple down sampling can be considered. However, if the input image has a lower resolution, it needs to be upscaled.
  - **Upscaling:** From the literature, DFDNet has been successfully employed to unblur and restore human face images. It is known as a deep face dictionary network for face restoration. DFDNet uses K-means to create deep dictionaries for key facial components from high-quality images. The proposed dictionary feature transfer (DFT) block matches and selects similar features from dictionaries, transferring high-quality details to degraded input. Component AdaIN reduces style diversity, and a confidence score adaptively fuses dictionary features. Multi-scale dictionaries enable progressive, coarse-to-fine restoration. Experimental results demonstrate the method’s effectiveness in realistic restoration of real degraded images without needing an identity-matching reference [24]. DFDNet was used to upscale low quality images to 1024x1024 to be fed to the model during the training process.
3. **All Levels of severity acneic face image generation**: Using StyleGAN2, this stage aims to generate synthetic acneic face images without distinguishing their severity level. In other words, the output of the StyleGAN2 can be either Mild, Moderate or Severe acneic face images. For this purpose, we consider a model pre-trained on celebrity faces (stylegan2-ffhq-config-f). Afterwards, a transfer learning approach is applied to update the last layer of StyleGAN2, considering the collected images (i.e. 1473 acneic face images) as input data. Transfer learning is widely used in deep learning because it allows training models from small datasets, decreasing the training time and the processing power [25].
4. **Mild/Moderate/Severe acneic face image generation**: the previous StyleGAN2 allows for generating an infinite number of composite synthetic acneic face images. This model might generate deformed images or images that present visual artifacts, these images are excluded from data gathering, examples are shown in Fig 4. Once cleaned and sorted into Mild, Moderate and Severe, three distinguished datasets are therefore obtained at this stage. Subsequently, three specific StyleGAN2 models are considered separately. First, the Mild acneic face image dataset is used as an input to a first pre-trained StyleGAN2 (from celebrity faces) to update the network’s last layer using transfer learning. This operation is repeated with Mild and Severe acneic face image datasets. Each StyleGAN2 is designed to generate one of the required cases.
5. **Hybrid synthetic-authentic classification**: in this stage, the aim is to design a hybrid synthetic-authentic classification system where the training phase is conducted by the synthetic generated images, including healthy face images, whereas the test phase employs authentic acneic face images. In other words, the input dataset merges five sub-datasets. Three of them are acquired from the output of StyleGAN2 models (Mild, Moderate and Severe), and one sub-dataset of health faces is generated with StyleGAN2. Finally, the last sub-dataset (i.e. Test phase purpose) contains only authentic acneic face images. For comparison and performance evaluation of the classification stage, we considered, in this study, three CNN-based synthetic-authentic classifier systems: InceptionResNetV2, ResNet152V2 and ResNet50V2. Four output classes are required: Healthy, Mild, Moderate, Severe and Healthy face images.

## 3 Results and discussion

We trained all our models on the NVIDIA DGX-1 server in the LISSI laboratory at UPEC. This server contains the following hardware:

- 8x NVIDIA Tesla^®^ V100 16 GB/GPU with 40960 Total NVIDIA CUDA^®^ Cores and 5120 Tensor Cores interconnected with NVIDIA NVLinkTM (1 gpu was used in the training)
- 2x 20-Core Intel^®^ Xeon^®^ CPU E5-2698 v4 2.2 GHz
- 512 GB DDR4 LRDIMM Memory
- 4X 1.92 TB SSDs RAID 0

### 3.1 StyleGAN2 performance evaluation

For evaluating our StyleGAN2 models, we used Fréchet Inception Distance as an evaluation metric Fréchet inception distance (FID) was introduced by Heusel et. al. in 2017 [26]. It calculates the similarity between synthetic and real images at a deep convolutional layer called InceptionV3. It does not compare images at pixel by pixel instead; it calculates the mean and standard deviation and it is defined as:

$$\begin{array}{c} FID={\left\|\mu_{r}-\mu_{g}\right\|}^{2}+T_{r}\left(\sum_{r}+\sum_{g}-2{\left(\sum_{r}\sum_{g}\right)}^{1/2}\right) \end{array}$$ (1)

- *μ*_*r*_ and *μ*_*g*_ real and generated images features’ means
- ∑_*r*_ and ∑_*g*_ are the variance matrices.
- *T*_*r*_ is the Trace operator.

**Fig 5:**
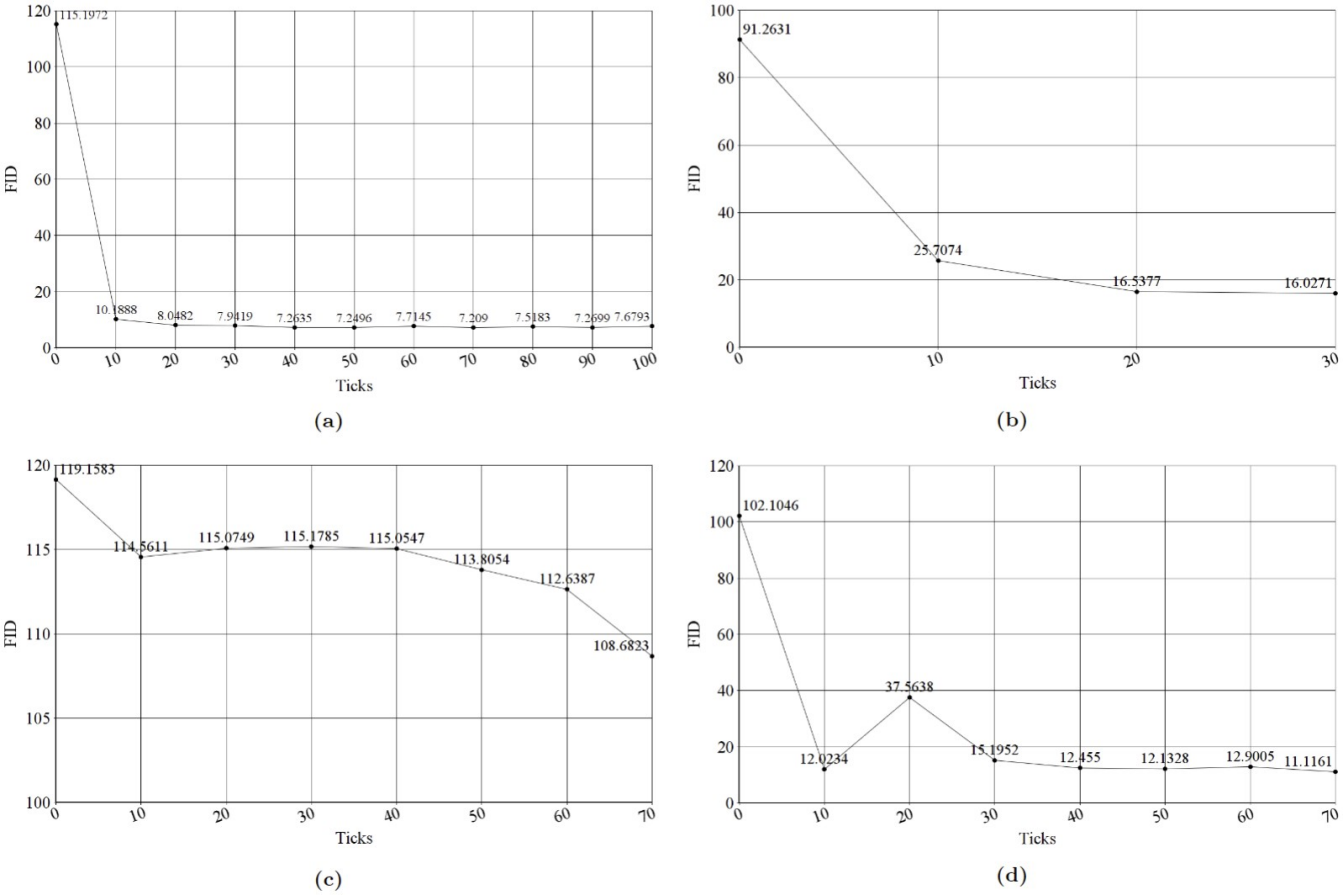
StyleGAN2 models FID graphs. (a) First Facial Acne Model FID Graph, (b) Mild Model FID Graph, (c) Moderate Model FID Graph, (d) Severe Model FID Graph.

**Fig 6:**
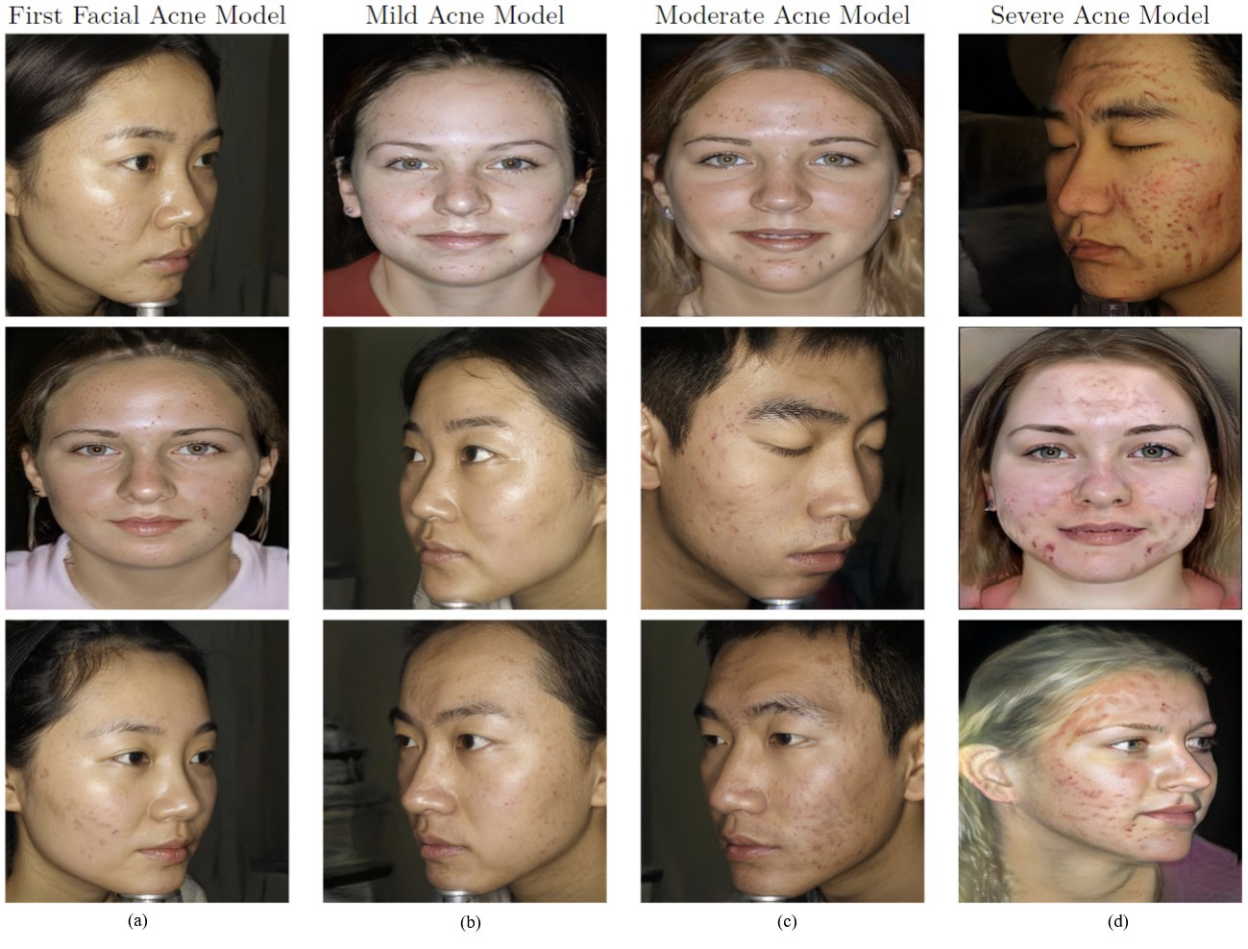
Samples from each model’s output. (a) Images generated from the first model (b) Images generated from mild acne model (c) Images generated from moderate acne model (d) Images generated from severe acne model.

After we finished training the first facial acne model and gathered 3 datasets of mild, moderate and severe facial acne images for training three additional StyleGAN2 models. We reached the results shown in Fig 5, each graph represents the FID of the model at each tick. first facial acne model (a) trained on 1473 real facial acne images, started with an FID of 115.1972 at tick 0 reaching the lowest of 7.209 at tick 70, training took 5d 17h 53m. Mild Acne Model (b) trained on 1500 generate facial acne images, started with an FID of 91.2631 reaching the lowest of 16.0271 at tick 30, training took 1d 18h 39m. moderate acne model (c) trained on 1502 synthetic images, was the least performing model reaching the lowest FID of 108.6823, training took 4d 13h 52m. Severe Acne Model (d) trained on 1514 synthetic images, started with and FID of 102.1046 reaching the lowest FID of 11.1161 at tick 70, training took 4d 00h 47m. All models were trained using the parameters shown in Table 1. Fig 6 contains samples generated by each StyleGAN2 model.

**Table 1:** Parameters used in training StyleGAN2 model, during training a snapshot of the model is saved every 10 ticks.

Parameter	Value
image-snapshot-ticks	10
mirror-augment	True
network-snapshot-ticks	10
D-lrate-base	0.002
G-lrate_base	0.002
minibatch-gpu-base	4
minibatch-size-base	32
rnd.np-random_seed	1000
total-kimg	25000

### 3.2 CNN models performance evaluation

For evaluating our CNN models we used the following metrics: accuracy, precision, recall and F1 score. Accuracy is the ratio of correctly classified samples over the total number of samples. Precision is the fraction of correctly classified positive samples over the total of positive samples. Recall is the fraction of positive samples over the total of correctly classified samples. F1 score is the harmonic mean between precision and recall [27].

$$\begin{array}{c} Accuracy=\frac{TP+TN}{TP+TN+FP+FN} \end{array}$$ (2)

$$\begin{array}{c} Precision=\frac{TP}{TP+FP} \end{array}$$ (3)

$$\begin{array}{c} Recall=\frac{TP}{TP+FN} \end{array}$$ (4)

$$\begin{array}{c} F1=2\times \frac{Precision\times Recall}{Precision+Recall} \end{array}$$ (5)

**Fig 7:**
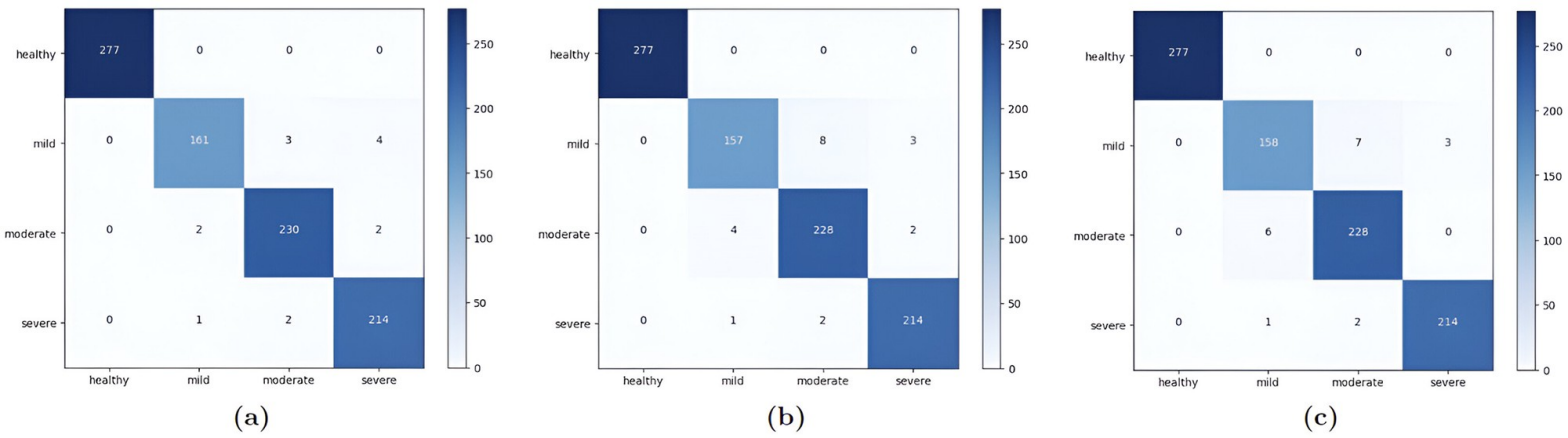
Confusion matrix for each CNN model on test split. (a) InceptionResNetV2 Confusion Matrix, (b) ResNet152V2 Confusion Matrix, (c) ResNet50V2 Confusion Matrix.

**Fig 8:**
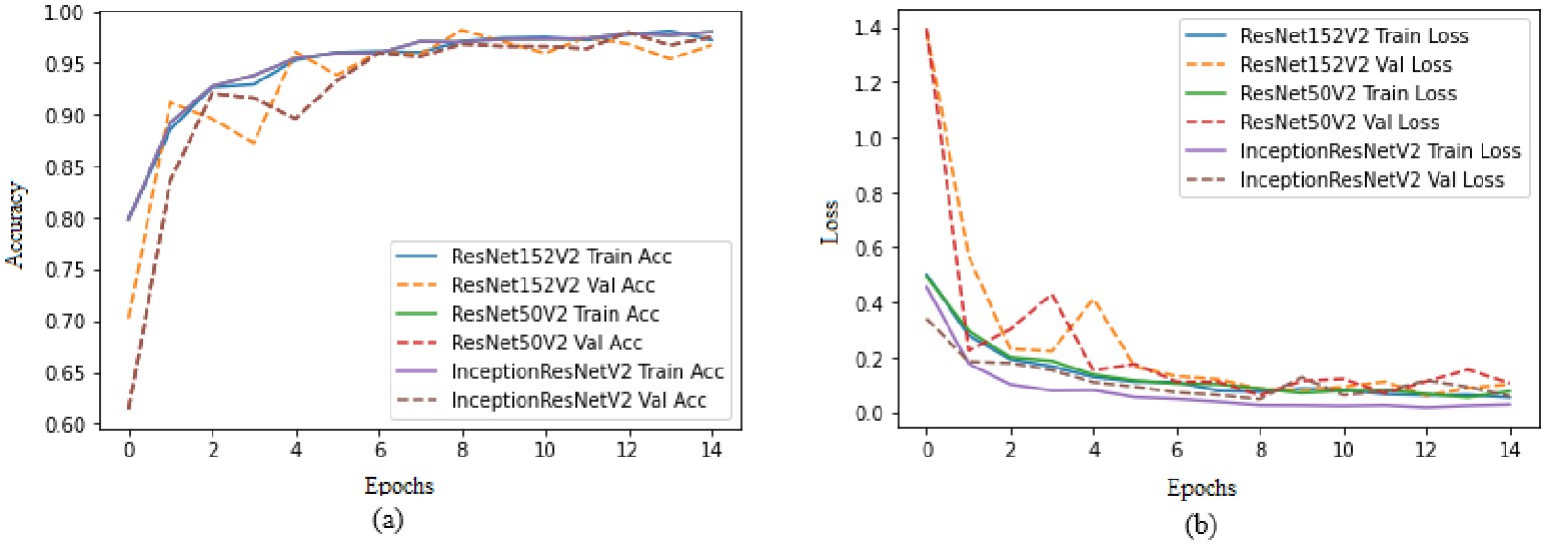
CNN models accuracy and loss graphs. (a) InceptionResNetV2, ResNet152V2 and ResNet50V2 models train and validation accuracy graphs, (b) InceptionResNetV2, ResNet152V2 and ResNet50V2 models train and validation loss graphs.

**Fig 9:**
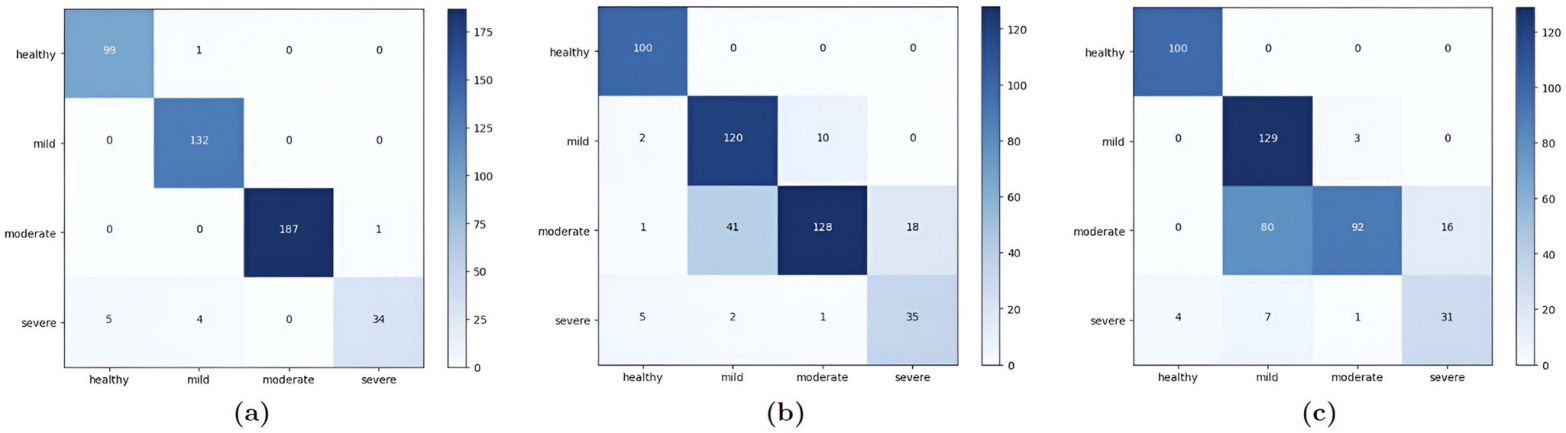
Confusion matrix for each CNN model tested on unseen images. (a) Confusion matrix of InceptionResNetV2: accuracy: 97.6%, (b) confusion matrix of ResNet152V2: ACCRUACY: 82.7%, (c) confusion matrix of ResNet50V2: accuracy: 76.03%.

**Fig 10:**
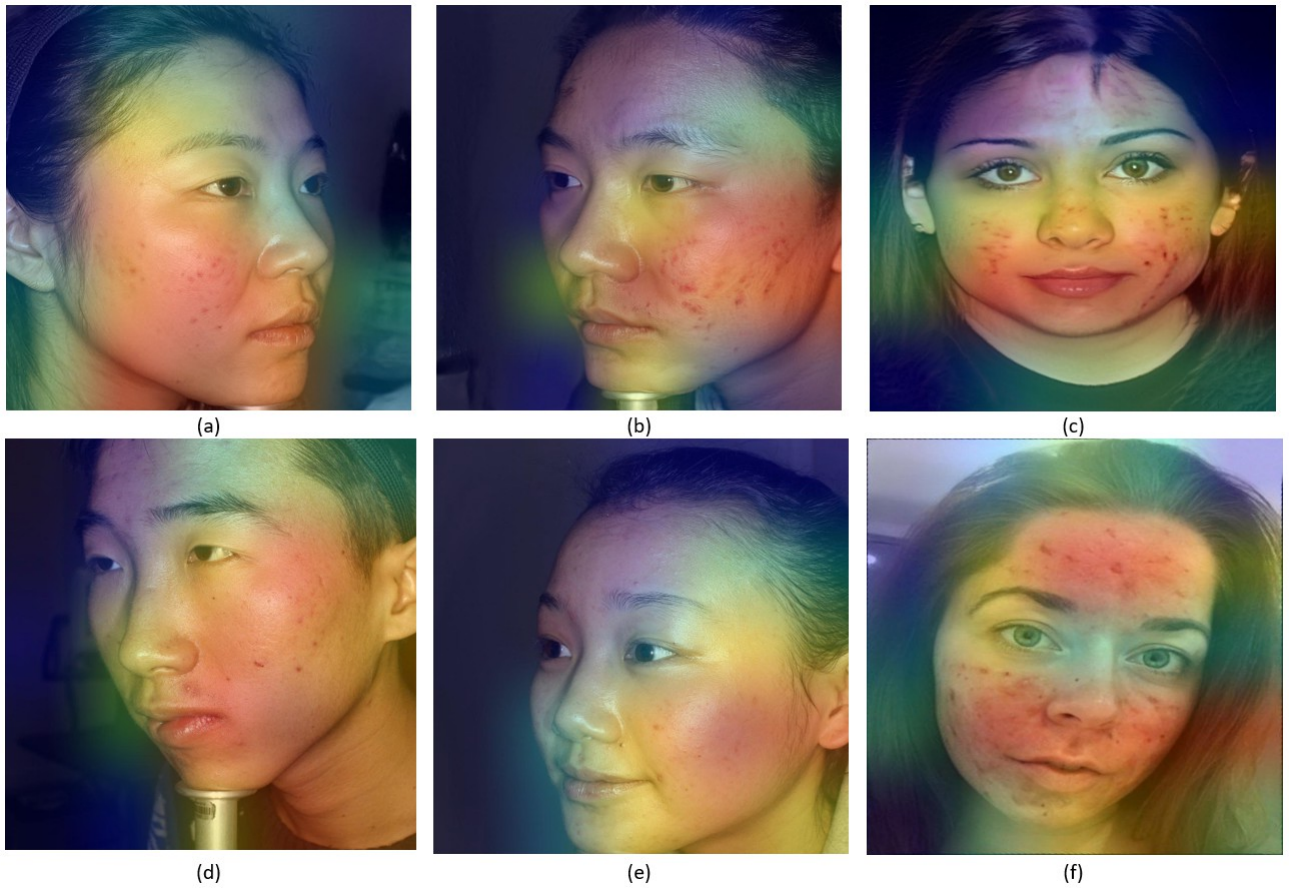
Gradcam representations for InceptionResNetV2 model. (a) Synthetic mild facial acne, (b) Synthetic moderate facial acne, (c) Synthetic severe facial acne, (d) Real mild facial acne, (e) Synthetic moderate facial acne, (f) Real severe facial acne.

In this paper we trained three CNN models using transfer learning on 4 classes of facial acne severity images using only synthetic images. The dataset was comprised of: 1387 healthy images, 841 mild Images, 1173 moderate images and 1086 severe images. Synthetic images generated from acne StyleGAN2 models were used in the train/test split, real images were used in the validation split. InceptionResNetV2 performed the best with an accuracy of 98.44%, precision 98.33%, 98.19% recall and 98.25% F1 score as shown in Table 2 and in Fig 7. Both loss and accuracy curves in Fig 8 are converging together showing no signs of overfitting. These results are not enough to prove that these models can classify facial acne correctly in real scenarios, therefor the evaluation of each model was done using unseen real facial acne images. Fig 9 shows that only InceptionResNetV2 (a) had a high accuracy of 97.6% and the other models struggled with classifying moderate class correctly. To prove the effectiveness of StyleGAN2 data augmentation, we trained another InceptionResNetV2 model on the same real facial acne dataset using the same parameters shown in Table 3, the only difference is that the second InceptionResNetV2 model uses Conventional augmentation (horizontal flip, brightness range, rotation) fed to the model in batches during training instead of StyleGAN2 augmentation. InceptionResNetV2-Conventional-Augmentation achieved an accuracy of 84.37% lower than InceptionResnetV2-StyleGAN2-Augmented 98.44% accuracy as shown in Table 4. Gradcam representations use the gradients of the last convolutional layer to visualize the regions of images that impacts the classification output the most and it uses a red to blue scale where red represents the most impactful region and blue the least. For additional validation of the InceptionResNetV2 model, Gradcam representations were used on both synthetic and real images to see how our model can detect acne correctly. Fig 10 shows gradcam representations on three synthetic images generated from our StylGAN2 models and three real facial acne samples. In all of the six images, the acne regions are highlighted in red whereas the rest of the face in yellow. the hair and background in each image was highlighted in blue meaning they had no impact on the classification result. These validation steps prove that InceptionResNetV2 trained on synthetic images is able to classify facial acne images correctly in real scenarios and that StyleGAN2 performs better in augmenting limited datasets compared to conventional augmentation methods. In addition, synthetic images generated using GAN models can be used in deep learning applications and give same results as real images. We have compared our work with existing research where generative adversarial networks data augmentation and convolutional neural networks skin disease classification were employed as shown in Table 5.

**Table 2:** CNN models performance evaluation using the following metrics: Accuracy, precision, recall and F1 score.

Model	Accuracy	Precision	Recall	F1 Score
InceptionResNetV2	**98.44%**	**98.33%**	**98.19%**	**98.25%**
ResNet152V2	97.77%	97.61%	97.38%	97.48%
ResNet50V2	97.88%	97.64%	97.53%	97.58%

**Table 3:** Parameters used in training CNN models. Model Checkpoint was used to save the model on each iteration if validation accuracy improves.

Parameter	Value
Batch Size	16
Epochs	15
Optimizer	ADAM
Learning Rate	0.0001
Classes	4
Total Images	4487
Train/Test Split	80%/20%
IMG_SIZE	224x224
Augmentation	Rotation/Brightness/Horizontal Flip
Model Checkpoint	True

**Table 4:** Comparison of InceptionResnetV2-StyleGAN2-Augmented and InceptionResNetV2-Conventional-Augmentation performance evaluation.

Model	Accuracy	Precision	Recall	F1 Score
InceptionResNetV2 + StyleGAN2 Augmentation	**98.44%**	**98.33%**	**98.19%**	**98.25**
InceptionResNetV2 + Conventional Augmentation	84.37%	74.41%	75.56%	74.49%

**Table 5:** Comparison of state-of-the-art techniques.

Authors	Technique	Dataset	Resolution	Objective
Junayed et. Al. (2019) [6]	AcneNet (CNN)	5 classes of acne disease with 360 images per class	224x224	Trained AcneNet CNN on 5 classes of acne achieving a 94% accuracy
Wu et. al. (2019) [7]	InceptionResNetV2 Models	6 classes of skin diseases with a total of 4006 training images and 388 test images		Comparison of multiple CNNs trained on a dataset of skin diseases. Their best achieving model (InceptionResNetV2) reached 77% average precision
Xiang et. al. (2020) [5]	ACGAN DenseNet-201	HAM10000 skin lesions dataset	64x64 upscaled to 256x256	Trained DenseNet201 on HAM10000 augmented with ACGAN (50% augmentation) and achieving a validation accuracy of 81.56%
Srinivasu et. al. (2021) [8]	MobileNetV2 LSTM	HAM10000 skin lesions dataset	224x224	Trained MobiletNetV2 and LSTM on HAM10000 dataset for mobile application classification of skin diseases. They achieved an accuracy of 85.34%
Zhao et. al (2021) [9]	Modified StyleGAN DenseNet201	ISIC2019 skin lesions dataset split into 20265 training images and 5066 validation images	224x224	Trained DenseNet201 on an augmented dataset using a modified stylegan and achieved a balanced multi-class accuracy of 93.64%
Proposed Method	StyleGAN2 InceptionResNetV2	ACNE04 Facial Acne Dataset	Low and High Resolution Images upscaled to **1024x1024** Resized dataset to 224x224 for CNN models training	Training of multiple StyleGAN2 models for generating synthetic images belonging to 3 levels of acne severity (mild, moderate, severe). In addition, trained multiple CNNs on a synthetic dataset of facial acne disease where the best performing model had **97.6%** validation accuracy on real facial acne images

### 3.3 Face manipulation detection

**Fig 11:**
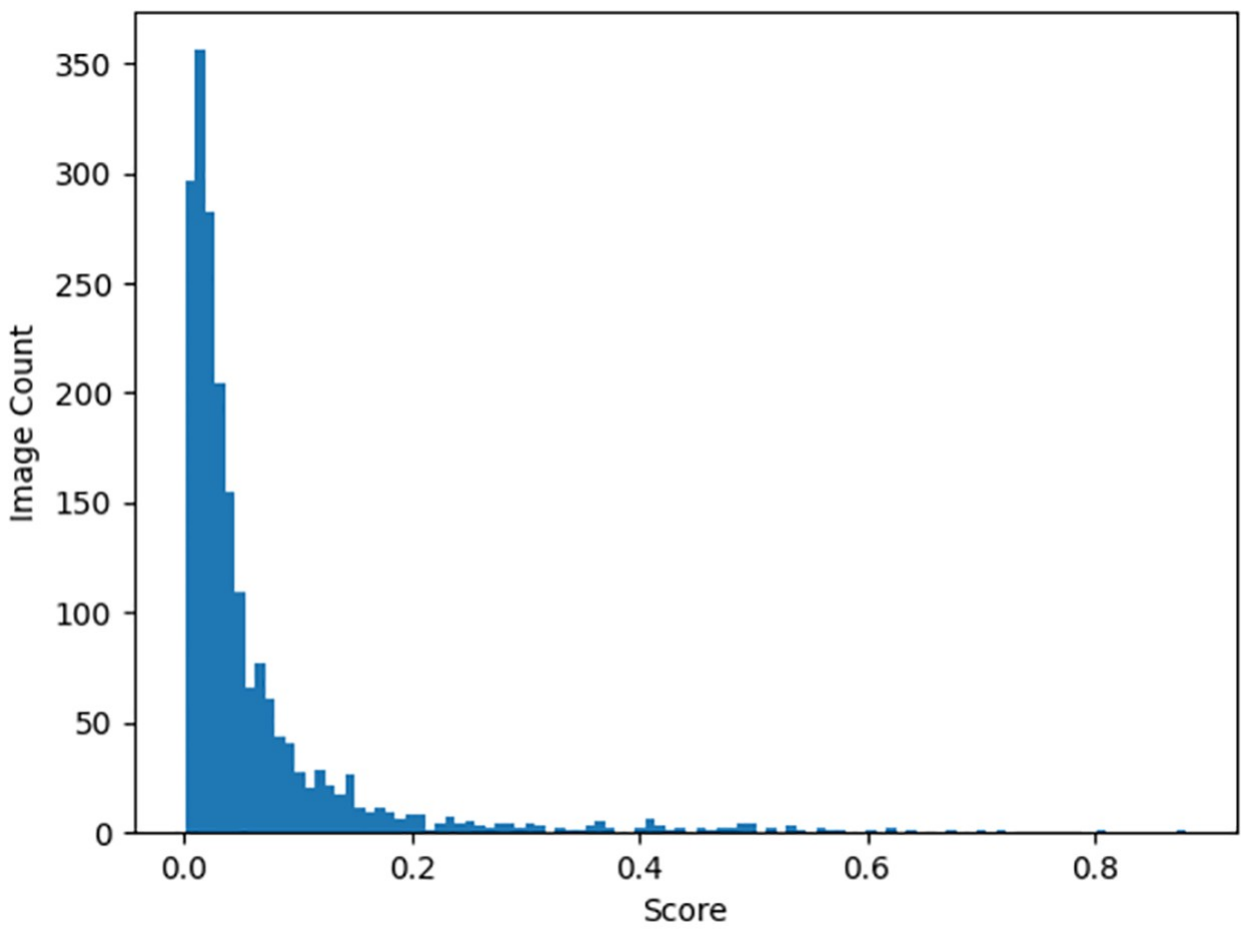
Distribution of synthetic facial acne images for each confidence score value ranging between 0 and 1.

Face manipulation detection is used to detect if faces in images or videos have been manipulated using deepfakes, faceswap or other methods. These methods can easily fool humans into thinking the manipulated images are authentic. Bonettini et. al. developed a face manipulation detection algorithm using an ensemble of CNNs tested on 100000 fake and 19000 real videos where each video has around 300 frames achieving top 3% ranking on this dataset. To further prove that our synthetic images are realistic, we used face manipulation detection EfficientNetB4 on 2000 synthetic facial acne images, this model takes an image on the input and outputs a confidence score between 0 and 1 where a value closer to 0 predicts real and closer 1 predicts fake [28]. After running the prediction we achieve these results: 98.9% of the images scored between 0 and 0.5 the majority being between 0 and 0.1 with 85%, on the other hand images that scored a value between 0.5 and 1.0 were only 1.1% of the total as shown in Table 6. The plot in Fig 11 represents the distribution of synthetic images for each confidence score.

**Table 6:** Predicted confidence score using face manipulation detection on a total of 2000 synthetic facial acne images.

Score Range	Count	Percentage
0.0-0.1	1700	85.0%
0.1-0.2	179	8.95%
0.2-0.3	52	2.6%
0.3-0.4	21	1.05%
0.4-0.5	26	1.3%
0.5-0.6	13	0.65%
0.6-0.7	6	0.3%
0.7-0.8	1	0.05%
0.8-0.9	2	0.1%
0.9-1.0	0	0.0%

### 3.4 Limitations

The dataset used presents the following limitations:

1. The individuals in the dataset belong the same ethnicity, which affects the output of the GANs that will generate acneic faces belonging to the same ethnicity.
2. The dataset contains 1473 images of real facial acne faces, a higher number of images is recommended to be used in validation which improves the performance of the CNN model in real-world scenarios.

## 4 Conclusion

This paper tackles the problem of the unavailability of free public dermatology datasets and their privacy. It offers a solution to help researchers and biomedical engineering communities to generate anonymous datasets for performance evaluation of their processing approaches. This study considered facial acne disease, an inflammatory skin disorder affecting around 9.4% of the population [29]. In our experiment, we collected 1473 images from multiple sources. These images were pre-processed (e.g. resized down or upscaled) using super-resolution deep-learning to generate 1024x1024 images and preserve the required information. A first StyleGAN2 model was trained using merged acneic face images corresponding to three levels of severity: mild, moderate and severe. Afterwards, at the next step, specific StyleGAN2 Models were used to generate each separately large-size dataset. The last phase of this work is to prove that training classifiers using synthetic acneic face images does not affect the classification performance when authentic acneic face images were used in the test phase. Moreover, we showed that the hybrid synthetic-authentic classifier based on InceptionResNetV2 achieved the best accuracy of 97.6%. A quantified metric is needed to represent the accuracy of the images, face manipulation detection model was used on 2000 synthetic images to classify them as as real/fake, 98.9% of the images scored less than 0.5 where 85% scored between 0 and 0.1 (closer to 0 is real, closer to 1 is fake). Finally, the proposed approach described in this paper allows the biomedical engineering community to generate unlimited synthetic and realistic images of acneic faces without facing privacy issues or the lack of datasets. In addition, dermatologists can also use these synthetic face images for educational purposes. Further work is needed to include more skin diseases with multiple levels of severity.
